# Broad environmental adaptation is associated with root anatomical phenotypes in maize landraces: an *in silico* study

**DOI:** 10.1093/aob/mcaf179

**Published:** 2025-08-13

**Authors:** Ivan Lopez-Valdivia, Harini Rangarajan, Miguel Vallebueno-Estrada, Jonathan P Lynch

**Affiliations:** Department of Plant Science, The Pennsylvania State University, University Park, PA 16802, USA; Department of Physiology and Cell Biology, Leibniz Institute for Plant Genetics and Crop Plant Research (IPK), OT Gatersleben, Seeland 06466, Germany; Department of Plant Science, The Pennsylvania State University, University Park, PA 16802, USA; Department of Plant Biology, University of Illinois Urbana-Champaign, Urbana, IL 61801, USA; The LatAmBio Initiative, Irapuato, Guanajuato 36821, Mexico; Department of Plant Science, The Pennsylvania State University, University Park, PA 16802, USA

**Keywords:** *OpenSimRoot*, root modelling, root phenotypes, maize landraces, *Zea mays* L. ssp. *mays*, local adaptation

## Abstract

**Background and Aims:**

Root phenotypes contribute to environmental adaptation. We hypothesized that root phenotypes of maize (*Zea mays* L. ssp. *mays*) landraces reflect their adaptation to edaphic limitations in their native soil environments, and that some root phenotypes may confer broad edaphic adaptation.

**Methods:**

We phenotyped the roots of maize landraces and used the functional–structural plant/soil model *OpenSimRoot_v2* to simulate landraces and their native environments to analyse how root phene states interact with each other and with environment variables to regulate edaphic adaptation.

**Key Results:**

Landraces from low phosphorus regions have root phenotypes with shallow growth angles and greater nodal root numbers, allowing them to adapt to their native environments by improved topsoil foraging. We used machine learning algorithms to detect the most important phenotypes responsible for adaptation to multiple environments. The most important phene states responsible for stability across environments are large cortical cell size and reduced diameter of roots in nodes 5 and 6. When we dissected the components of root diameter, we observed that large cortical cell size improved growth by 28, 23 and 114 %, while reduced cortical cell file number alone improved shoot growth by 137, 66 and 216 %, under drought, nitrogen and phosphorus stress, respectively. Functional–structural analysis of 96 maize landraces from the Americas, previously phenotyped in mesocosms in the glasshouse, suggested that parsimonious anatomical phenotypes, which reduce the metabolic cost of soil exploration, were the main phenotypes associated with adaptation to multiple environments, while root architectural phenotypes were related to adaptation to specific environments.

**Conclusions:**

These results indicate that integrated root phenotypes with anatomical phene states that reduce the metabolic cost of soil exploration increase tolerance to edaphic stress across multiple environments and therefore would improve yield stability, regardless of their root architecture.

## INTRODUCTION

The development of crops with greater tolerance to abiotic stress and reduced input requirements is an important goal for global agriculture (Lynch, 2019, 2022; Lynch *et al*., 2024). Improving edaphic resource acquisition by targeted selection of root phenotypes is an opportunity to adapt crops to degraded soil environments (Lynch *et al*., 2022). The inclusion of root phenotypes into crop breeding programmes is challenging due to the complexity of the fitness landscape and phenotyping constraints (Burridge *et al*., 2019). However, Burridge *et al*. (2019) show a successful case where root phenotypes were deployed in breeding programmes to significantly improve common bean (*Phaseolus vulgaris* L.) yield in Mozambique. A better understanding of the fitness landscape of root phenotypes will facilitate the deployment of root phenotypes in breeding programmes for more resilient crops.

The functional–structural plant/soil model *OpenSimRoot_v2* (*OSRv2*) dynamically simulates root growth in three dimensions, soil resource distribution and acquisition, and root–soil interactions (Schäfer *et al*., 2022). *OSRv2* and its predecessors *OpenSimRoot* (Postma *et al*., 2017) and *SimRoot* (Lynch *et al*., 1997) allow the simulation of diverse root phenotypes and environments, permitting analysis of myriad root–soil interactions (Rangarajan *et al*., 2022). For example, *SimRoot* predicted that root cortical aerenchyma (RCA) would improve plant performance in low-fertility soils (Postma and Lynch, 2011), which was then confirmed in field experiments (Saengwilai *et al*., 2014*a*; Chimungu *et al*., 2015; Galindo-Castañeda *et al*., 2018). Also, *SimRoot* predicted that reduced lateral root branching frequency would improve plant performance in low-nitrogen soils but that greater lateral root branching frequency would improve plant growth in low-phosphorus soils (Postma *et al*., 2014; Rangarajan *et al*., 2018), which was later confirmed in field experiments (Zhan *et al*., 2015; Jia *et al*., 2018). It also predicted that the ‘Three Sisters’ or ‘*milpa*’ polyculture of maize (*Zea mays* L. ssp. mays), common bean and squash (*Cucurbita pepo* L.) would have greater below-ground niche complementarity than the respective monocultures (Postma and Lynch, 2012), which was subsequently confirmed in the field (Zhang *et al*., 2014). More recently, *OSRv2* capabilities have expanded to include more robust simulation of photosynthesis, responses to water deficit (Schäfer *et al*., 2022) and soil mechanical impedance (Strock *et al*., 2022; Rangarajan and Lynch, 2024), making it useful for the analysis of root–soil interactions in multiple environments.

The ideal integrated phenotype for a particular environment, or ‘ideotype’, is a function of the circumstances (Rasmusson, 1987). For instance, independent field experiments showed that increased nodal root number (Sun *et al*., 2018), high lateral branching frequency (Jia *et al*., 2018), increased root hair formation (Zhu *et al*., 2010*a*; Miguel *et al*., 2015), reduction in root metabolic cost (Lynch, 2015; Galindo-Castañeda *et al*., 2018) and shallow axial root growth angles (Zhu *et al*., 2005; Miguel *et al*., 2015) improved the colocalization of roots with phosphorus-rich soil domains, improving crop performance under low phosphorus availability, confirming the utility of the ‘*Topsoil Foraging*’ ideotype (Lynch and Brown, 2001). In contrast, independent field experiments showed that steep axial root angles (Trachsel *et al*., 2013; Schneider *et al*., 2022), fewer nodal roots (Saengwilai *et al*., 2014*b*; Gao and Lynch, 2016), few laterals (Zhan *et al*., 2015; Zhan and Lynch, 2015) and reduction of the metabolic cost of soil exploration (Zhu *et al*., 2010*b*; Jaramillo *et al*., 2013; Chimungu *et al*., 2014*a*, 2014*b*; Saengwilai *et al*., 2014*a*) improve subsoil foraging, and hence improve plant performance in low-nitrogen and drought environments, confirming the utility of the ‘*Steep, Cheap and Deep*’ ideotype (Lynch, 2013). While the ‘*Topsoil Foraging*’ and the ‘*Steep, Cheap and Deep*’ root ideotypes are optimized for specific edaphic environments, they exhibit trade-offs for deep and shallow soil resources, and it would be useful to gain a better understanding of root phenotypes that confer broad environmental adaptation.

It has been proposed that genotypes that react less to the environment are needed to guarantee consistent crop production in a range of environments (Francis and Kannenberg, 1978). Most yield stability studies have tested several hybrids in multiple environments and normally particular hybrids are presented as the most stable (e.g. Fan *et al*., 2007; Shiri, 2013; Zhao and Yang, 2018), but the factors underlying yield stability are not well understood. It has been proposed that yield stability is associated with increased stress tolerance (Tollenaar and Lee, 2002). On the other hand, it has been demonstrated that soil environments that reduce stress, for instance by increasing soil water-holding capacity, are associated with yield stability (Williams *et al*., 2016). More recent studies have shown that optimal fertilization (Knapp and van der Heijden, 2018; Ahrends *et al*., 2021) often results in greater yield stability. Previously, we described how specific combinations of root anatomical and architectural phenotypes improve soil exploration in specific edaphic environments, improving plant performance (Lynch, 2022; Lynch *et al*., 2024). However, it is unknown if integrated root phenotypes that increase tolerance to edaphic stress would also contribute to yield stability.

Root architectural phenotypes that colocalize roots with soil resource availability improve plant performance in soils with suboptimal water, nitrogen and phosphorus availability (Lynch, 2019, 2022), while root anatomical phenotypes that reduce the metabolic cost of soil exploration will improve plant performance under nitrogen, water and phosphorus stress (Lynch *et al*., 2021). Therefore, we hypothesize that anatomical phenotypes that reduce the metabolic cost of soil exploration and intermediate architectural phenotypes will be broadly adapted to multiple environments. In this study we used laser ablation tomography (LAT) and *shovelomics* to characterize the anatomy and architecture of the roots of eight maize landraces in the field. We used *OSRv2* to reconstruct the root phenotypes and their native soil and atmospheric environments *in silico*. We evaluated their performance and used machine learning models to determine the most relevant phene states associated with environment-specific and broad adaptation. We constructed synthetic root phenotypes designed for environment-specific and overall optimal plant performance. Furthermore, we extended our analysis to 96 maize landraces, previously phenotyped by Burton *et al*. (2013), by simulating their growth in the eight environments using *OSRv2* and evaluating the most important phenotypes for broad adaptation. Finally, we evaluated the components of the synthetic phenotypes independently to determine their contributions to drought, nitrogen and phosphorus stress.

## MATERIAL AND METHODS

### Model description

*OSRv2* is a functional–structural plant/soil model that simulates root growth in three dimensions, soil nutrient distribution and root–soil interactions (Postma *et al*., 2017; Schäfer *et al*., 2022). Root architecture is simulated as a hierarchical development of root classes, branching frequencies, growth vectors and growth rates. *OSRv2* includes a carbon module that calculates the available carbon for growth based on a subtraction of the carbon sources minus the carbon sinks. The carbon sinks are the root exudates and the maintenance cost due to respiration, while the carbon sources are the carbon available in the seed and the carbon produced by photosynthesis. Carbon partitioning rules determine that exudates and respiration are obligate costs, and once this demand is covered the remaining carbon could be used for growth. The model considers constraints to root and shoot growth due to the availability of resources such as nitrogen, phosphorus and water (Schäfer *et al*., 2022), but also due to soil impedance (Strock *et al*., 2022). The shoot is simulated by several state variables in a non-geometrical fashion (Schäfer *et al*., 2022). Responses to water deficit are simulated considering the Farquhar–von Caemmerer–Berry model for photosynthesis, leaf gas exchange and stomatal conductance, leaf temperature and energy balance models, sun/shade model for leaf-to-canopy scaling, a nitrogen-limited photosynthesis model, water stress response functions and models for day/night cycles (Schäfer *et al*., 2022), making it sensitive to fluctuations in atmospheric conditions such as CO_2_, temperature and solar radiation among others. The movement of water in the soil is simulated by the SWMS3D model (Simunek *et al*., 1995). The radial and axial conductivity of water in the roots is simulated by a hydraulic network model (Alm *et al*., 1992; Doussan *et al*., 1998). Evapotranspiration is simulated with the Penman–Monteith equations (Penman, 1948; Monteith, 1965). Soil hydraulic properties, root water uptake, precipitation and evaporation will determine the soil water dynamics (Schäfer *et al*., 2022). Nitrogen movement in the soil is also simulated by the SWMS3D model. Nitrogen mineralization is simulated by the Yang and Janssen model (Yang and Janssen, 2000). To simulate plant responses to stress the model calculates stress factors based on the optimal resource concentration in the tissue versus the actual resource acquisition. The stress factors will directly impact leaf expansion rate and light use efficiency. When the leaf expansion rate is constrained, the sink of the shoot is reduced, and proportionately more carbon is allocated to the roots, functioning as a growth regulator. On the other hand, when light use efficiency is affected, there is less carbohydrate availability and a reduction of growth in general. The impact of stress factors on leaf expansion rate and light use efficiency is resource-specific (Postma and Lynch, 2011; Schäfer *et al*., 2022).

### Accession selection

We selected eight maize (*Zea mays* L. subsp. *mays*) accessions from a root phenotypic diversity panel (Burton *et al*., 2013) from the most extreme environments in terms of aridity and humidity (Supplementary Data Table S1): four 4 accessions from humid regions (Costa Rica [PI471823], Cusco, Peru [PI571629], Huánuco, Peru [PI571913], Madre de Dios, Peru [PI571994]), and four accessions from arid regions (Mendoza, Argentina [PI516002], Lambayeque, Peru [PI571439], Ica Peru [PI571523], Colorado, USA [PI608619]). Arid regions also coincided with soils having low nitrogen availability but high phosphorus availability, while humid regions tend to have soils with greater nitrogen availability but low phosphorus availability. The location of every accession was obtained from georeferenced information available at USDA, Agricultural Research Service, National Plant Germplasm System (2024). We obtained soil nitrogen, phosphorus and precipitation data from SoilGrids (Poggio *et al*., 2021), a dissertation by Jaramillo-Velasteguí (2011) and POWER Data Access Viewer v.2.0.0 (https://power.larc.nasa.gov/data-access-viewer/ consulted on 15 September 2023), respectively.

### Root parameterization

#### Architecture

We grew 10 plants per accession at the Russell E. Larson Agricultural Research Farm at Rock Spring, PA, USA (40°42′40.915″N, 77°57′11.120″W) from June through September 2021. We fertilized using 150 kg N ha^−1^ (urea) to fulfil the optimal requirements for maize informed by preplant soil testing. We provided drip irrigation as needed. Planting was performed in single-row plots at a density of 73 300 plants ha^−1^. After 90 d of growth, all plants were sampled following the *shovelomics* procedure (Trachsel *et al*., 2011). Roots were washed and processed for subsequent phenotyping. Root phenotyping consisted of imaging the root system while sequentially removing a set of nodal roots per node. This process was repeated in every node, producing an image for every node until we removed all the nodal roots. Once we removed all nodal roots, we took a final image of the seminal roots and counted the seminal root number manually. We used ImageJ (Sosa *et al*., 2014) to analyse the images. For every node, we measured the angle, nodal root number, lateral branching frequency and root diameter. The root angle was measured considering the horizon as the starting point. For every accession, we integrated their root phenotypic data per node into *OSRv2* using the input file production script available at: https://github.com/ilovaldivia/LopezValdivia_2024_YieldStability.git.

#### Anatomy

To phenotype root anatomy we sampled 4-cm root segments, 5–9 cm from the base of a second-nodal root for all plants and stored them in 70 % (v/v) ethanol in water. We chose second nodal roots for consistent sampling since maize nodal roots increment their diameter from older to younger nodal positions (York and Lynch, 2015; Yang *et al*., 2019). Another consideration for choosing the second nodal root is that they have consistent responses to drought compared to other nodal roots (Koehler *et al*., 2025) and have proved to be a representative sample in root anatomy studies, including root metabolic cost studies (Lopez-Valdivia *et al*., 2023; Sidhu and Lynch, 2024), root microbial associations (Galindo-Castañeda *et al*., 2019), and root phenotypic diversity in landraces and wild relatives (Burton *et al*., 2013). Furthermore, selecting the second node allowed us to expand our analysis to several landraces from the Americas using a public dataset from Burton *et al*. (2013) as described in the ‘Functional analysis of maize populations of the Americas’ section. We imaged three cross-sections along the 4-cm root segment utilizing LAT (Strock *et al*., 2019). LAT consists of an oscillating pulsed laser (s-Pulse HP, 343 nm THG, Amplitude Systems) that segments the root sample while a camera (α7R III digital camera, Sony; MP-65 mm F/2.8 Lens photo macro 1–5, Canon) gathers cross-sectional images of the ablated root surface. We measured cortical cell size (CCS), cortical cell file number (CCFN) and RCA using RootScan 2.4v (https://plantscience.psu.edu/research/labs/roots/methods/computer/rootscan). We used a multiple linear regression model reported by Lopez-Valdivia *et al*. (2023) to predict respiration and nitrogen construction cost per gram of root tissue using CCS and CCFN as predictors. We integrated CCS and CCFN into *OSRv2* as root respiration and nitrogen construction cost predictions, while RCA was integrated directly.

### Environment parameterization

We designed eight environments in *OSRv2* corresponding to the origin of each accession. To design the environments, we obtained soil and atmospheric parameters from SoilGrids (Poggio *et al*., 2021) and POWER Data Access Viewer v.2.0.0 (https://power.larc.nasa.gov/data-access-viewer/ consulted on 15 September 2023), respectively. For the atmosphere, we extracted data for wind speed (WS), minimum (T_MIN) and maximum (T_MAX) temperature, precipitation (PRECIP), and relative humidity (RH) in a period from 1980 to 2021 (Supplementary Data S1). For each atmospheric parameter, we used an average of the 1980–2021 period, and we selected 60 d corresponding to a growing season considering the maize planting dates in each region (Data S2). For the soil, we used soil core information from SoilGrids to extract nitrogen concentration, soil texture, bulk density and soil organic matter. The closest core to the coordinate of origin was considered a representative of the soil environment. Soil core IDs and soil core coordinates are available in Data S3. We used the soil texture to calculate the van Genuchten parameters (Data S3) utilizing Rosetta3 (https://www.handbook60.org/rosetta/). We inferred the soil taxon from the USDA taxonomy system using soil texture information and FAO classification, and we obtained the average phosphorus concentration for that soil class from Jaramillo-Velasteguí (2011). We integrated the soil and atmospheric data into *OSRv2* with the input file production script available at: https://github.com/ilovaldivia/LopezValdivia_2024_YieldStability.git.

### Model runs

To test the performance of the eight accessions across the eight environments we ran 64 simulations of all accession/environment combinations in *OSRv2* (Schäfer *et al*., 2022) with git v.9621150b6f. To test the performance of synthetic phenotypes we ran 72 simulations for nine synthetic phenotypes (eight environment-specific and one general) under eight environments. We ranked the accessions based on shoot biomass, 1 being the greatest biomass and 8 the least. Furthermore, we ran 12 simulations for the dissected phenotypes which included the independent effect of branching frequency (BF), CCS, and root diameter of nodes 5 and 6 (Dia), as well as combinations of them (BF + CCS; BF + Dia; CCS + Dia). A reference root phenotype with contrary values of BF, root diameter and CCS compared with the general synthetic was simulated for comparison proposes. Simulations were run utilizing High-Performance Computing at Pennsylvania State University. All input files required to replicate the simulations can be found at: https://github.com/ilovaldivia/LopezValdivia_2024_YieldStability.git. All data were extracted and analysed using R v.4.2.3 (R Core Team, 2019).

### Synthetic phenotypes

To identify the most important root phenotypes in each environment and across all environments we ran a Boruta feature selection package (Kursa *et al*., 2010) in R v.4.2.3 (R Core Team, 2019). We used the most important root phenotypic parameters for each environment to build a multiple regression model using R v.4.2.3 (R Core Team, 2019). We used the coefficients of the multiple regression to modify the variables that were selected in Boruta. For instance, if the coefficient was positive for the branching frequency for node 4, we would increase the value of the synthetic phenotype to the maximum value available in the distribution of the eight maize accessions. We repeated the process for all relevant phenotypes in all environments to create the specific synthetic phenotypes. To create the general synthetic root phenotype, we repeated the process using the average of all the environments.

### Functional analysis of maize populations of the Americas

To explore the functional utility of maize root phenotypes across the Americas we analysed root anatomy and architecture data available from Burton *et al*. (2013) using a combination of machine learning algorithms and *OSRv2*. We selected the data only for landraces which had geo-localization data. We performed a principal component analysis and K-means clustering to find the main clusters of phenotypes using R Studio v.4.2.3 (R Core Team, 2019) and the MASS package (Venables and Ripley, 2002). To evaluate a correlation between geographical location and the root phenotypic clusters, we compared the latitude variances among the clusters using one-way ANOVA and Tukey’s significance test. We classified the main clusters as categorical variables and we built a linear discriminant model to predict the cluster category based on environmental data including precipitation, elevation, nitrogen soil concentration and phosphorus soil concentration. Finally, we used *OSRv2* to simulate root growth corresponding to the clusters of root phenotypes identified by the principal component analysis in the eight environments described earlier in the ‘Environment parameterization’ section. Simulation data were analysed and graphed using R Studio v.4.2.3 (R Core Team, 2019) and the ggplot2 package (Wickham, 2016).

## RESULTS

### Variation of root phenotypes across multiple nodes

**Fig. 1.**
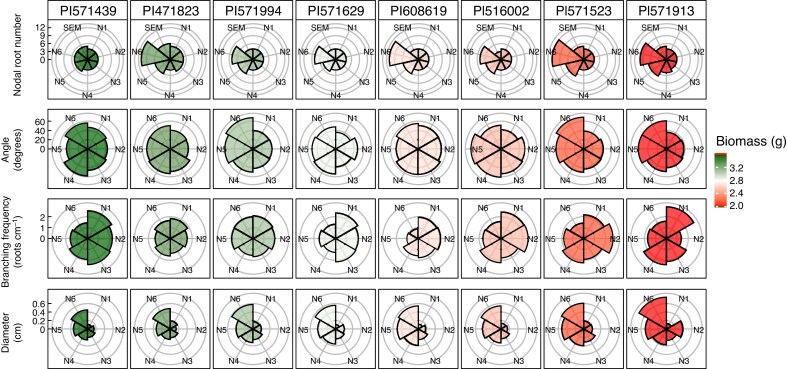
Parameterization of the root system architecture of eight maize landrace accessions from the Americas. Accessions are organized from left to right from the greatest to the least average shoot biomass across the eight environments. All measurements were taken by node and ‘N’ represents the node number. SEM represents seminal roots.

Root phenotypes display wide variation among open-pollinated populations (e.g. Burton *et al*., 2013; Lynch, 2022). We observed no variation in the number of nodal roots in nodes 1, 2 and 3, while significant variation was observed for nodes 4, 5 and 6 (Fig. 1). Root anatomy showed less variation for cortical cell file number (8–12) and percentage of aerenchyma (5–25 %) compared with the phenotypic ranges observed by Burton *et al*. (2013) in 195 landraces from the Americas (6–15 and 6–37 % for cortical cell size and percentage of aerenchyma, respectively). However, we observed different phenotypic variation when compared with data from inbred populations. For instance, we observed that cortical cell size in landraces (393–1434 µm) was generally larger than in inbred IBM populations (100–900 µm^2^) as reported in Chimungu *et al*. (2014*a*) and Lopez-Valdivia *et al*. (2023). Overall, our root phenotypic measurements represent sufficient phenotypic variation for the purposes of this study.

### Non-stable accessions showed contrasting strategies for soil exploration

**Fig. 2.**
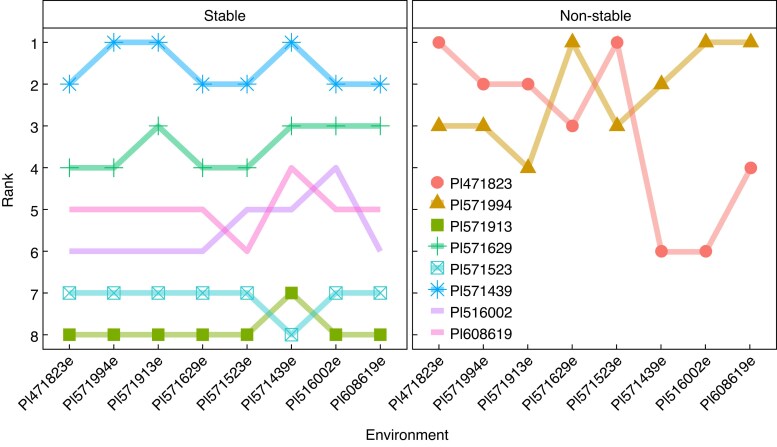
Simulated reciprocal transplanting of eight maize landraces in eight environments. Accessions are divided into two groups corresponding to their pattern across all environments: stable (PI571629, PI571439, PI571913, PI516002, PI608619) and non-stable (PI471823, PI571994). Rankings were based on shoot biomass, 1 being the greatest biomass and 8 being the least biomass.

**Fig. 3.**
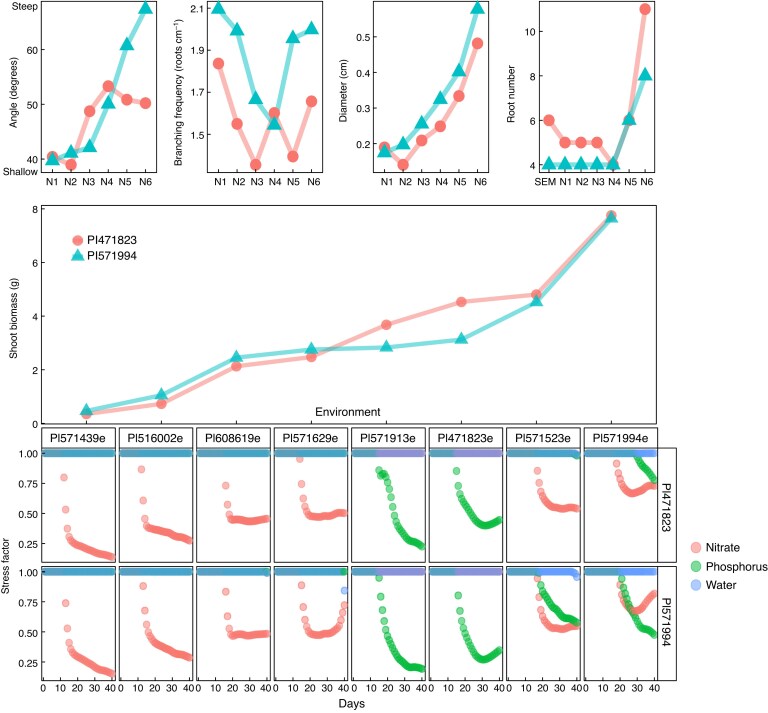
Non-stable maize landraces have contrasting integrated root phenotypes. PI571994 has steeper angles, less branching frequency, thicker root diameter and fewer nodal roots compared with PI471823. Top panel: root phenotypes per node (‘N’ represents the node number and ‘SEM’ represents seminal roots). Middle panel: shoot biomass in the eight environments. Bottom panel: nitrate, phosphorus and water stress factors in the soil in a 40-d simulation, where stress factor values closer to 0 represent greater stress.

The spatiotemporal colocalization of roots and soil resources will determine resource uptake and its utility in a particular environment. To explore the utility of each of the eight integrated phenotypes we reconstructed their root phenotypes and environments in *OpenSimRoot* and simulated a reciprocal transplanting (we will refer to the native environments as the accession name in italics plus the letter ‘e’ at the end, e.g. *PI571629e*). When ranking the accessions based on shoot biomass, we observed that the rankings of six accessions were stable across all environments. In contrast, two accessions (PI471823 and PI571994) ranked between the first or second best in some environments but then shifted to fourth to sixth place in other environments. We classified the consistent and non-consistent ranking accessions as stable and non-stable, respectively (Fig. 2). To analyse the differences between the non-stable accessions we compared their root phenotypes and evaluated their performance in contrasting environments. Accession PI471823 has shallower root angles in younger nodes, greater number of nodal roots and reduced lateral root branching frequency compared with PI571994 (Fig. 3), including some of the phenotypes of the ‘topsoil foraging’ ideotype (Lynch and Brown, 2001). Accession PI471823 also had greater shoot biomass compared with accession PI571994 in environments *PI571629e* and *PI471823e*, which showed the greatest levels of phosphorus stress. On the other hand, accession PI571994 had steeper angles, a reduced number of nodal roots and increased lateral branching frequency (Fig. 3). Steeper root growth angles and reduced production of nodal roots are elements of the ‘steep, cheap and deep’ ideotype (Lynch, 2013). The simulation of the root phenotype of PI571994 also produced greater biomass in environments *PI571439e*, *PI516002e*, *PI608619e* and *PI571629e*, which have the greatest nitrogen stress. Overall, the non-stability of accessions PI471823 and PI571994 is due to their extreme root phenotypes, especially regarding nodal root number and root growth angle, improving their adaptation to specific environments but making them unstable across all the environments.

### Phene states associated with greater stability include large cortical cell size

**Fig. 4.**
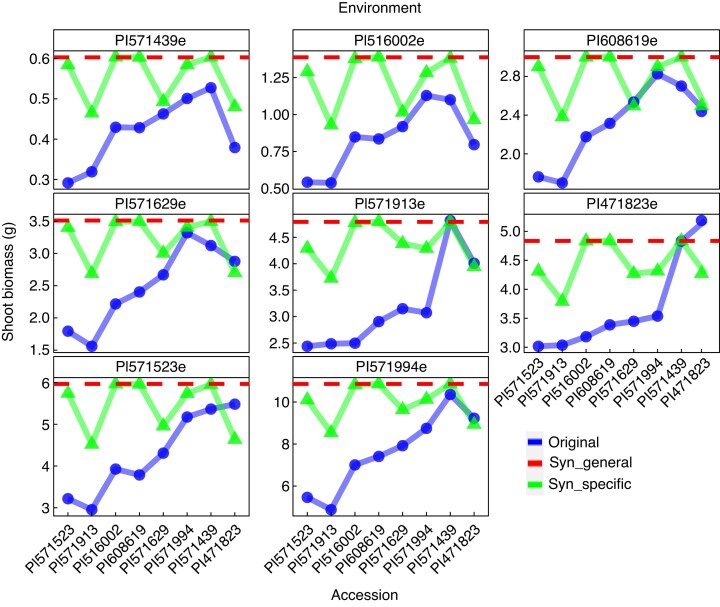
Shoot biomass of synthetic root phenotypes designed for specific and general environments. Environments are organized from top left to bottom right from the least to the greatest shoot biomass production. The blue line (Original) represents the original root phenotype for each accession. The green line (Syn_Specific) represents the specific synthetic phenotype that was optimized for its native environment. The red line (Syn_general) represents the general synthetic phenotype that was optimized for all environments. Synthetic phenotypes are described in Table 2.

To understand root phenotypes contributing to stability across environments we employed a Boruta feature selection algorithm to find the most important phenotypes for each environment and across all environments. In general, lateral branching frequency in node 4, cortical cell size, and root diameter of nodes 5 and 6 were important across all environments (Table 1). Every environment had a specific combination of phenotypes that were important. For instance, in environments *PI471823e* and *PI571913e*, root diameter is the most important variable determining biomass (Table 1), while in environments *PI516002e* and *PI571439e*, nodal root number and cortical cell size are the most important variables determining biomass (Table 1). To understand if an optimized root phenotype for a specific environment would surpass a root phenotype optimized for multiple environments, we designed synthetic phenotypes for specific and general environments using multiple linear regression on the root phenotypes selected previously by Boruta (a detailed description of the synthetic phenotypes design is available in the Methods). We observed that the general synthetic root phenotype produced greater biomass compared with the original phenotypes in six of eight environments (Fig. 4). Most of the specific synthetic phenotypes were superior to their original (Fig. 4), except for accession PI471823. However, the general synthetic root phenotype produced greater biomass than five of the specific synthetic phenotypes. The synthetic phenotypes customized for environments *PI516002e*, *PI608619e* and *PI571439e* had similar performance as the general synthetic phenotype (Fig. 4). The general synthetic phenotypes and synthetic phenotypes customized for environments *PI516002e*, *PI608619e* and *PI571439e* share a large cortical cell size, which is probably the factor driving their superior performance. The general synthetic phenotype had the best performance across environments (Fig. 4). Synthetic phenotypes generated for their specific environments outperformed the actual phenotype from that environment in almost all cases (Fig. 4).

**Table 1.** Feature selection with Boruta of the root phenotypes that are important in every environment and across all environments (General).

	Environment								
Rank	*471823e*	*516002e*	*571439e*	*571523e*	*571629e*	*571913e*	*571994e*	*608619e*	General
1	Dia_N5	NRN_N8	NRN_N8	Dia_N5	Dia_N5	Dia_N5	Dia_N5	BF_N4	BF_N4
2	Dia_N4	NRN_N6	NRN_N6	CCS	BF_N4	Dia_N6	Dia_N4	NRN_N5	CCS
3	Dia_N6	CCS	NRN_N7	Dia_N4	Dia_N4	Dia_N4	BF_N4	CCS	Dia_N5
4	Dia_N3	NRN_N7	CCS		NRN_N5	Dia_N7	CCS	Dia_N5	Dia_N6
5		AN_N7	BF_N4		Dia_N3	Dia_N3			

Dia, root diameter; NRN, nodal root number; AN, root angle; BF, lateral branching frequency; N#, node number; CCS, cortical cell size.

### Evaluation of individual components of synthetic phenotypes

**Fig. 5.**
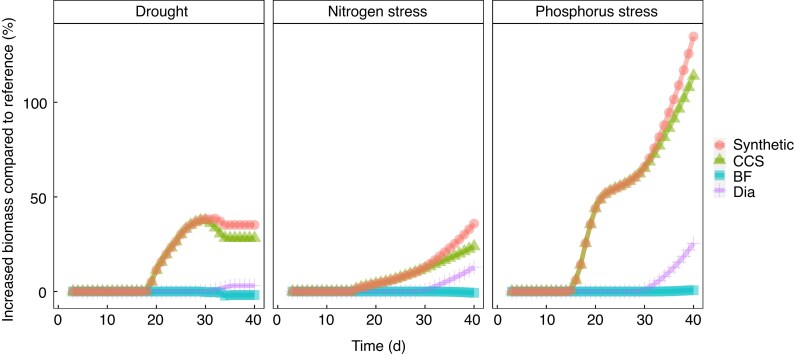
Evaluation of each phene state of the synthetic phenotype under drought in isolation (left panel), nitrogen stress in isolation (centre) or phosphorus stress in isolation (right panel). The ‘Synthetic’ phenotype represents all phene states simulated together. CCS represents the independent effect of large cortical cell size, BF represents the independent effect of reduced lateral branching frequency, and Dia represents the independent effect of small root diameter of nodes 5 and 6.

To understand the individual contributions of each phene state to the general synthetic phenotype we simulated the effect of each phene state alone and evaluated its performance compared with a reference phenotype. The effect of reduced root diameter in nodes 5 and 6 (Dia) improved shoot growth 3, 13 and 19 % compared with the reference, in drought, nitrogen and phosphorus stress conditions, respectively (Fig. 5). Large CCS improved biomass 28, 23 and 114 % compared with the reference phenotype in drought, nitrogen and phosphorus stress, respectively. The combined effect of large CCS and smaller diameter (synthetic) showed an additive effect by improving shoot growth 35, 33 and 134 % compared with the reference under drought, nitrogen and phosphorus stress, respectively (Fig. 5). We observed a minor increase in biomass of decreased branching frequency of axial roots of node 4 of 0.54 and 0.4 % compared with the reference in nitrogen and phosphorus stress, respectively. Decreased branching frequency of axial roots of node 4 decreased plant biomass by 1.86 % under drought. Overall, the greatest effect of the synthetic phenotypes is given by large CCS and reduced diameter of nodes 5 and 6, while the effect of branching frequency in node 4 is irrelevant.

### Functional characterization of root phenotypes of maize landraces

#### Principal component analysis and K-means clustering of root phenotypes

**Fig. 6.**
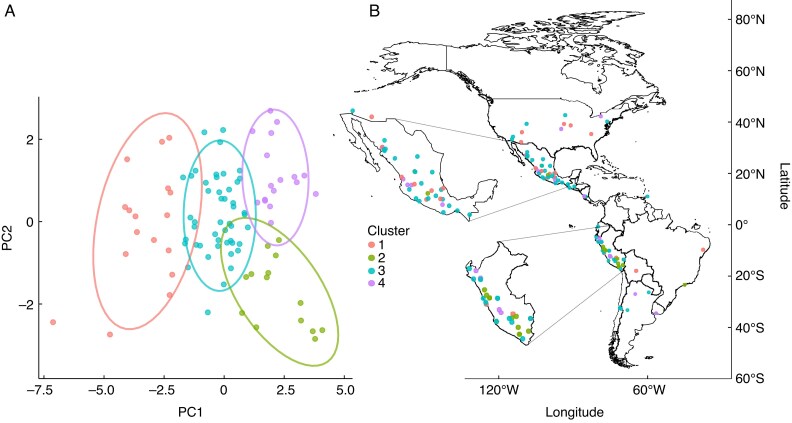
Principal component analysis and K-means clustering of root phenotypes from 100 maize landraces of the Burton *et al*. (2013) panel. (A) K-means clustering in four main groups. (B) Geographical distribution of maize landraces across the Americas.

**Fig. 7.**
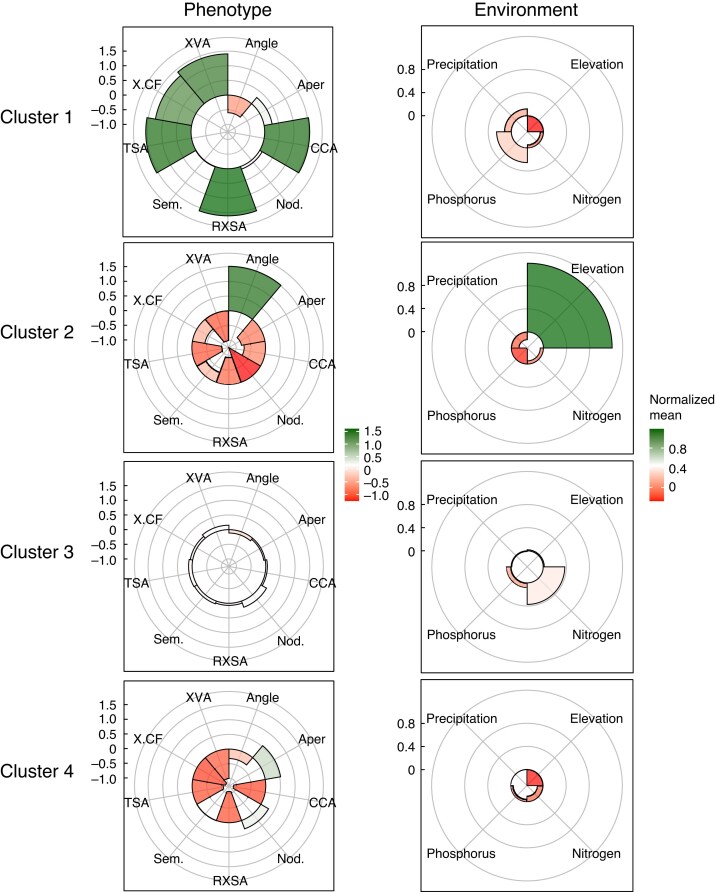
Normalized values of root phenotypes and environmental factors describing the four clusters identified by principal component analysis and K-means clustering. Root phenotype abbreviations correspond to ‘XVA’: xylem vessel area, ‘X.CF’: cortical cell file number, ‘TSA’: total stele area, ‘Sem.’: seminal root number, ‘RXSA’: root cross-section area, ‘Nod.’: nodal root number, ‘CCA’: cortical cell area, and ‘Aper’: percentage of aerenchyma.

To capture greater phenotypic diversity in our analysis we performed a principal component analysis and K-means clustering in a maize diversity panel (Burton *et al*., 2013) to find clusters of root phenotypes. We found four main clusters (Fig. 6A) distributed across different regions of the Americas (Fig. 6B). Clusters 1 and 2 showed significant differences in their spatial distribution in the Americas (Table 2; Fig. 6B). Cluster 1 was distributed mostly in North America, while cluster 2 was mostly distributed in the Andean highlands of South America (Figs 6B and 7). The root phenotypes grouped in cluster 1 were characterized by having shallow nodal growth root angle and thick nodal roots with large anatomical phenotypes (e.g. large xylem vessels, large stele area, increased cortical cell file number and increased cortical cell area; Fig. 7). In contrast, root phenotypes grouped in cluster 2 tend to have steeper root growth angles, fewer nodal roots and thin nodal roots with smaller anatomical phenotypes (e.g. small xylem vessel area, reduced cortical cell file number, small stele area and reduced cortical cell area; Fig. 7). Clusters 3 and 4 were distributed evenly across the Americas. The root phenotypes grouped in cluster 3 were characterized by having an average phenotype (Fig. 7), while root phenotypes classified in cluster 4 tend to have a greater number of thin nodal roots with shallow growth angle and small anatomical phenotypes (e.g. small xylem vessel area, small stele area, reduced cortical cell file number and small cortical cell area; Fig. 7).

**Table 2.** Analysis of variance and Tukey test of the importance of latitude among four clusters identified by PCA and K-means.

Cluster	Difference	lwrLower	Upper	*P* adj
2-1	−24.2663	−44.3459	−4.18667	0.011212
3-1	−7.12959	−22.8323	8.573106	0.635918
4-1	−15.5939	−34.6772	3.489367	0.148837
3-2	17.13668	0.23718	34.03618	0.045577
4-2	8.672351	−11.4072	28.75195	0.671965
4-3	−8.46433	−24.167	7.238367	0.496121

Comparison of cluster means are shown in the cluster column. For instance, 2-1 refers to the comparison of cluster 1 and cluster 2 means.

#### Linear discriminant analysis captures environmental variables

Clusters 1 and 2 were distributed in North and South America, while clusters 3 and 4 were distributed evenly across the Americas. To understand the environmental variables associated with each cluster, we performed a linear discriminant analysis using clusters 1–4 as categories and environmental variables as predictors. Cluster 1 was described mainly by lowlands in North America with less availability of nitrogen, but greater availability of phosphorus and precipitation compared with the other clusters (Fig. 7). Cluster 2 was described by the highlands of South America, with low availability of phosphorus and nitrogen, and reduced precipitation (Fig. 7). Cluster 3 was described by mid-altitude and relatively abundant nitrogen conditions. Cluster 4 was described by lowlands and average conditions for phosphorus, nitrogen and precipitation (Fig. 7). Our model predicted the cluster category in the test dataset with 58 % accuracy. Phenotypes grouped in cluster 1, with shallow and thick nodal roots, come from low nitrogen environments, while phenotypes grouped in cluster 2, with fewer and steep nodal roots, tend to have reduced precipitation and low-phosphorus soils.

#### Functional analysis of root phenotypes using *OpenSimRoot*

**Fig. 8.**
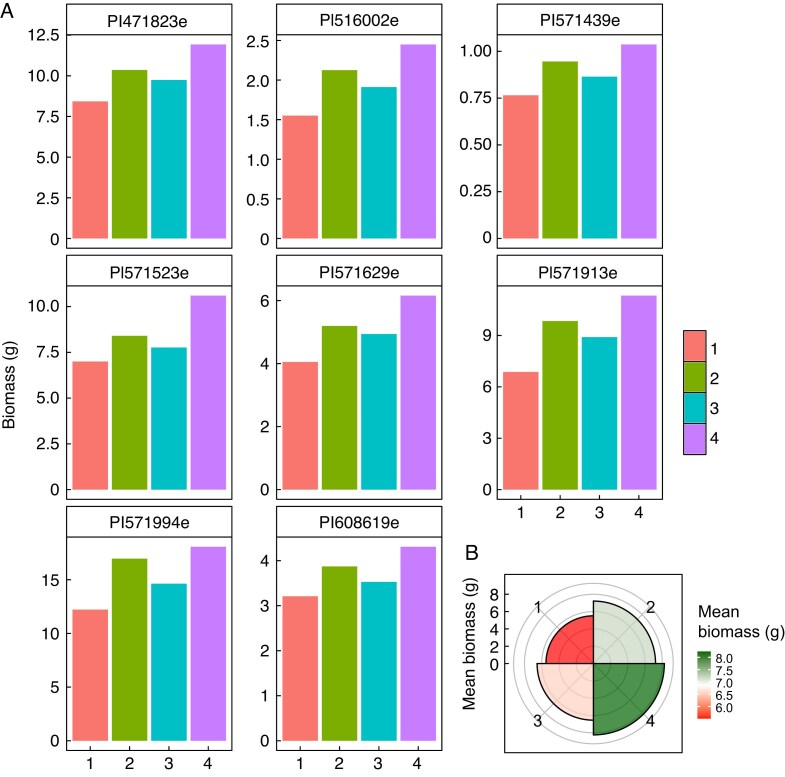
Simulation of root phenotypes of clusters 1–4 under eight different environments of the Americas. (A) Simulated shoot biomass of the four clusters in every environment. The environment name corresponds to the location of origin from the eight accessions previously evaluated. (B) Mean shoot biomass of the eight environments for the root phenotypes corresponding to clusters 1, 2, 3 and 4.

**Fig. 9.**
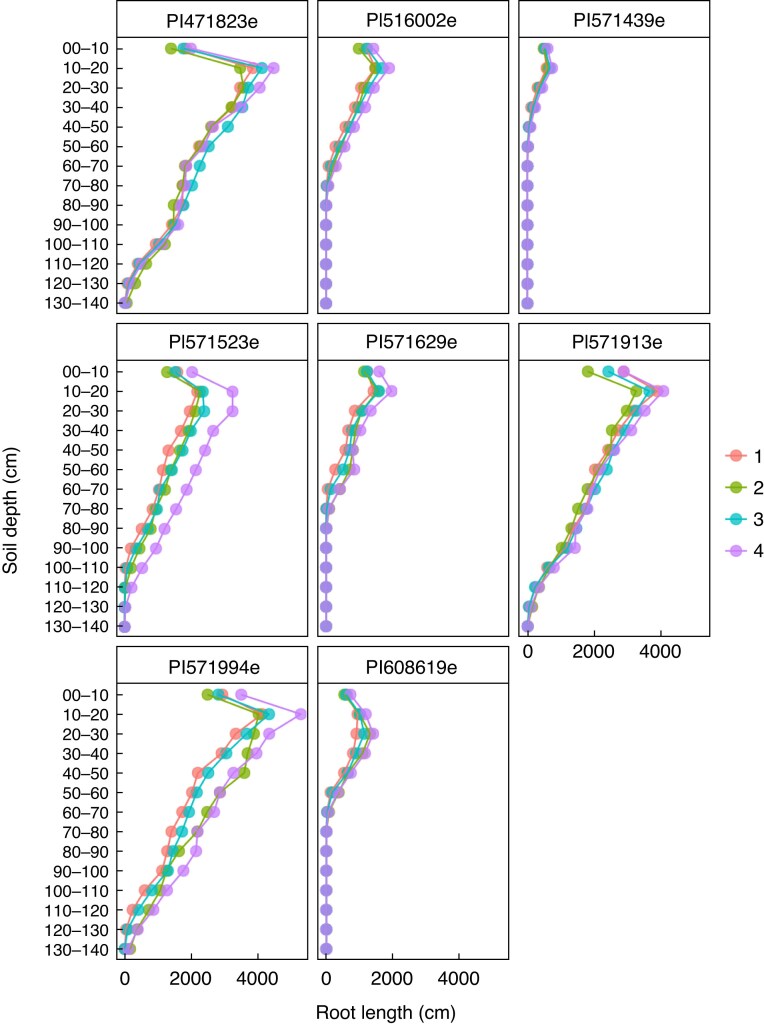
Simulated root depth of clusters 1–4 in eight different environments. The root depth of simulations at day 40 are shown. The panel name corresponds to the environment.

**Fig. 10.**
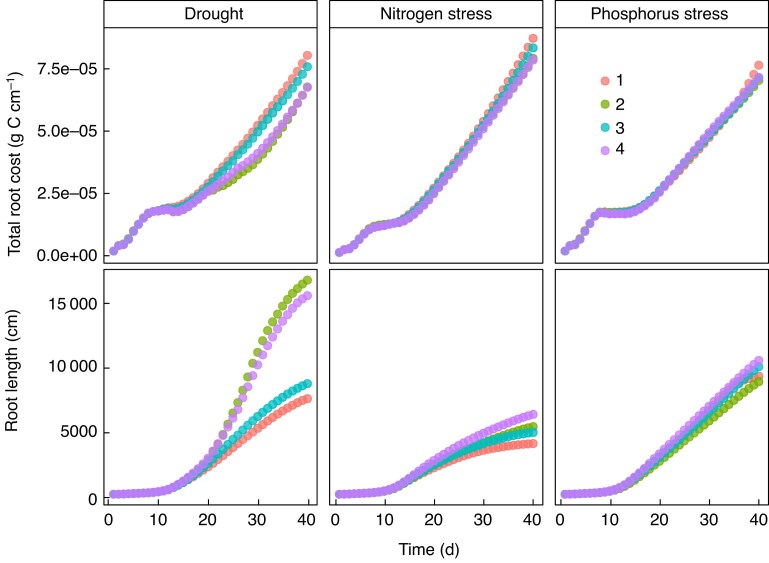
Comparison of total root carbon cost per centimetre of root length and total root length over time of phenotypic clusters 1–4 under drought, nitrogen and phosphorus stress. Different colours indicate the cluster number.

**Fig. 11.**
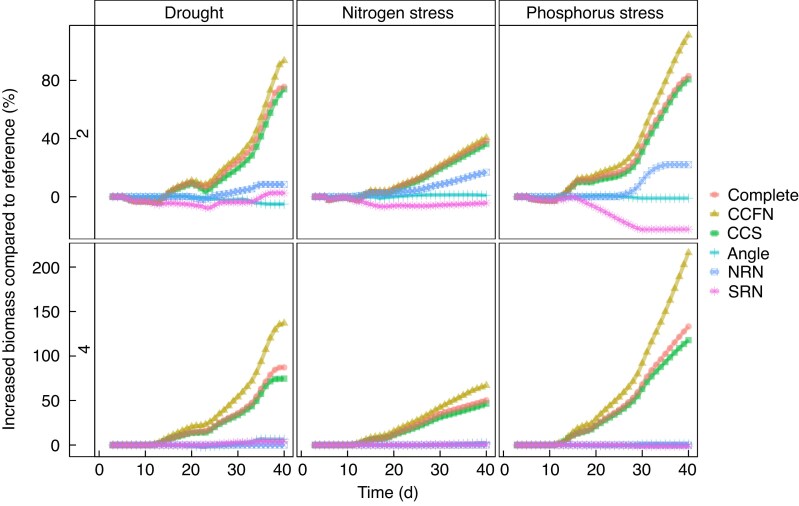
Phenotypic dissection of the root phenotypes of clusters 2 and 4 (top and bottom panel). The phenotypes of clusters 2 and 4 were deconstructed into nodal root growth angle (Angle), cortical cell file number (CCFN), cortical cell size (CCS), nodal root number (NRN) and seminal root number (SRN). Simulations correspond to 40 d. The phenotype of cluster 3 was used as a reference phenotype.

To understand the functional utility of the integrated phenotypes grouped in clusters 1–4, we simulated the root growth of each cluster into eight contrasting environments using *OSRv2*. We observed that the root phenotype representing cluster 4 produced greater biomass in all the environments compared with the other groups (Fig. 8). The cluster 4 phenotype was characterized by roots with small diameter but with many nodal roots and shallow growth angles. The second best was the root phenotype representing cluster 2, which also produced small-diameter roots, but with reduced number of nodal roots and steeper growth angles. Interestingly, the phenotype of cluster 4 showed a dimorphic system with significantly greater root length in the shallow and deeper soil layers (Fig. 9), surpassing the root depth of the steep-angle phenotype of cluster 2. This can be explained by the shallow angles which promote a distribution of the root length in the topsoil, and by reduced root diameter, which reduces the metabolic cost of roots and promotes a deeper distribution of root length. This can be visualized when we compare the total carbon cost and root length of clusters 2 and 4. The total root carbon cost of cluster 4 is less than that of cluster 2, which produces an increased root length (Fig. 10). We dissected the root phenotypes of clusters 2 and 4 to understand which variables contributed to their improved performance. For cluster 2, reduced cortical cell file number alone improved shoot growth by 94, 41 and 111 % under isolated drought, nitrogen and phosphorus stress (Fig. 11). For cluster 4, reduced cortical cell file number improved shoot growth by 137, 66 and 216 % under isolated drought, nitrogen and phosphorus stress (Fig. 11). On the other hand, we observed that small cortical cell size reduced biomass compared to the complete phenotype, indicating that the main effect of parsimonious anatomical phenotypes comes from reduced cortical cell file number instead of cortical cell size. The contribution of other elements such as angle, nodal root number and seminal root number was irrelevant in cluster 4 (Fig. 11). However, in cluster 2, reduced nodal root number contributed 8, 17 and 22 % as the increment in biomass compared to the reference (cluster 3), under drought, nitrogen and phosphorus stress, respectively. Cluster 1, which was characterized by having thick roots with steeper angles, had higher root carbon cost (Fig. 10), reduced root depth (Fig. 9) and reduced shoot biomass (Fig. 8). Cluster 3, the average phenotype, occupied the third place in production of biomass among the four groups. In general, clusters 2 and 4, which had a reduced root diameter and reduced cost of soil exploration, were the best performers in all environments regardless of the architecture, suggesting that phenotypes that reduce the metabolic cost are essential for broad environmental adaptation.

## DISCUSSION

We explored root phenotypic variation of maize landraces from the Americas and simulated their performance across multiple environments to understand how integrated root phenotypes contribute to stability across environments. We found that non-stable accessions with steep roots and fewer nodal root numbers produced greater biomass in environments where nitrogen is the main stress factor, while accessions with shallower roots and increased number of nodal roots had better performance in environments where phosphorus was the limiting factor. Boruta feature selection showed that the most important phenotypes associated with greater biomass production across all environments are small diameters in nodes 5 and 6, large cortical cell size, and reduced lateral branching frequency in node 4. We evaluated the independent effects of these phene states and found that large cortical cell size alone improved growth by 28, 23 and 114 %, while reduced cortical cell file number alone improved shoot growth by 137, 66 and 216 %, under drought, nitrogen and phosphorus stress, respectively. We extended our analysis to 96 maize landraces, previously phenotyped by Burton *et al*. (2013), by simulating their growth with *OSRv2* and showed that having small anatomical phenotypes improved plant growth across all environments regardless of architectural traits such as root growth angle and number of nodal roots.

### Cheaper phenotypes offset the cost of expensive, but useful, phenotypes

Independent studies have shown that shallower root angles (Zhu *et al*., 2005; Miguel *et al*., 2015; Lynch, 2022) and an increased number of nodal roots (Sun *et al*., 2018) colocalize roots in the phosphorus-rich topsoil, improving plant performance under phosphorus stress. In contrast, steeper root growth angles (Trachsel *et al*., 2013; Schneider *et al*., 2022) and reduced number of nodal roots (Saengwilai *et al*., 2014*a*) colocalize roots in nitrogen-rich subsoil domains and improve plant performance under nitrogen stress. Our results showed that non-stable accessions developed extreme phenotypes, for instance shallow angles and a large number of nodal roots for PI471823, and steeper root angles with fewer nodal roots for PI571994. Our simulations showed that PI471823 is better adapted to environments with phosphorus stress, while PI571994 is better adapted to environments with nitrogen stress (Fig. 3). Both accessions come from humid regions with relatively mild nitrogen stress (Costa Rica [PI471823], Madre de Dios, Peru [PI571994]). However, Costa Rica soils have 0.289 ppm available phosphorus while Madre de Dios soils have 0.649 ppm available phosphorus (Jaramillo-Velasteguí, 2011). Since Costa Rican soils have low phosphorus bioavailability, it is logical that PI471823 presents root phenotypes optimized for phosphorus stress. It is interesting that the synthetic phenotypes with broad adaptation were characterized by having anatomical phenotypes that reduce the metabolic cost of soil exploration, while synthetic phenotypes for specific environments were characterized by anatomical phenotypes but also root architectural phenotypes such as nodal root number and root angle. This indicates that anatomical phenotypes that reduce root metabolic cost improve plant performance regardless of the environment, while root architectural phenotypes provide a more environmental-specific adaptation. Our results are consistent with previous theoretical studies (Dathe *et al*., 2016; Rangarajan *et al*., 2018) and with empirical results observed in bean genotypes by Strock *et al*. (2019) where integrated phenotypes were associated with adaptation to low fertility and drought.

It is interesting that despite the fact that most of the synthetic phenotypes performed better than their original phenotypes, accession P1471823 performed better than the synthetic in its own environment. To explain this, we need to analyse the native environment of P1471823, the phenotype of the native accession, and the ‘optimal’ phenotype suggested by Boruta. Environment *P1471823e* corresponds to Costa Rica (9°53′55.3″N 84°08′11.4″W), a region with high abundance of Andosols, which are soils of volcanic origin with low phosphorus bioavailability. As discussed in the Introduction, several studies have shown that maize genotypes that have a shallow nodal root growth angle (Zhu *et al*., 2005; Miguel *et al*., 2015), increased number of nodal roots (Sun *et al*., 2018), increased seminal root number (Zhu *et al*., 2006; Perkins and Lynch, 2021) and high lateral branching frequency (Postma *et al*., 2014; Jia *et al*., 2018) function as an integrated phenotype that improves plant performance under suboptimal phosphorus bioavailability, also known as the ‘topsoil foraging’ ideotype (Lynch and Brown, 2001). The root phenotype of accession P1471823 combines shallow nodal root growth angles, more nodal roots and more seminal roots (Fig. 3), which aligns with the ‘topsoil foraging’ ideotype and explains its superior performance in a low-phosphorus environment. Interestingly, the Boruta algorithm selected the diameter of nodes 5, 4, 6 and 3 as the most important features for adaptation to the environment *P1471823e* and neither nodal root number, seminal root number nor root angle were selected as important features. This illustrates that, even though Boruta feature selection is capable of identifying the most important phene states conferring adaptation to a particular environment, in some cases, important phenotypes could be neglected by the model and researcher expertise should be involved to complement potential gaps. It is possible that there is not enough phenotypic variation in the eight landraces used for this study and the Boruta algorithm is not able to find a strong signal of these phenotypes, and therefore a larger dataset to train Boruta would also improve its ability to predict the best phenotypic combinations for particular environments. Nevertheless, if we intend to select a stable phenotype for multiple environments, the highly specialized phenotypes, such as the one exhibited by accession P1471823, would probably be avoided given its poor performance in environments different from its own.

Structure–function analysis of 96 landraces showed that clusters 1 and 2 were preferentially distributed in North and South America, respectively, while clusters 3 and 4 were distributed broadly between the Americas. This reinforces the hypothesis that there are stable and non-stable root phenotypes, where the non-stable phenotypes are restricted to specific environments, while the stable phenotypes are more broadly adapted to a range of environments. The simulations of clusters 1–4 in multiple environments showed that cluster 2 (specifically from South America) and cluster 4 (broadly distributed in the Americas) were the best performers under all environments even though they showed completely contrasting root architectures. It is interesting that the clusters of stable (broadly adapted) root phenotypes were both broadly distributed in the Americas in one case (cluster 4) and coming from a particular region in another case (cluster 2). On the other hand, the non-stable (environment-specific) root phenotypes were also both broadly distributed in the Americas in one case (cluster 3) and were region-specific in another case (cluster 1). This suggests that the stability of a root phenotype is not restricted to their environmental origin, indicating that root phenotypes that provide broad adaptation, such as anatomical phenotypes that reduce the metabolic cost of soil exploration, might appear equally by inheritance from previous populations locally adapted to their conditions, but also as a result of convergent evolution. However, maize landraces migrate constantly to different regions of the Americas due to seed dispersal by farmers and traders, producing a potential mismatch between their genetic relationships and their geographical distribution. Phylogenetic studies that integrate root phenotypes will be required to understand this phenomenon in more detail. Overall, our results showed that the broad adaptation of root phenotypes is not restricted to the environmental origin and might have also been the result of convergent evolution.

It has been proposed that yield stability is associated with tolerance to stress (Tollenaar and Lee, 2002). Several long-term studies have shown that yield stability increased in fields that had optimal fertilization (Knapp and van der Heijden, 2018; Ahrends *et al*., 2021). Our results showed that the most important phene states conferring adaptation to a wide range of environments are large cortical cell size and reduced diameter of roots in nodes 5 and 6. Several studies have shown that large cortical cell size reduces the metabolic cost of soil exploration and improves plant adaptation to nitrogen stress (Lopez-Valdivia *et al*., 2023), phosphorus stress (Sidhu and Lynch, 2024) and drought (Chimungu *et al*., 2014*a*). Our results suggest that phenotypes that reduce the metabolic cost of soil exploration and improve plant response to stress are also associated with yield stability. It is important to note that root diameter is an aggregate phenotype composed of multiple elemental phenes such as cortical cell file number, cortical cell size and stelar phenes. Cortical cell diameter is a better predictor of the energy cost of the root compared with root diameter as it considers the living cortical area (Jaramillo *et al*., 2013; Colombi *et al*., 2019). Additionally, a functional–structural analysis of 96 landraces showed four main clusters of root phenotypes. Our results showed that clusters 2 and 4, with a small root cross-section area (RXSA), reduced stele area (TSA), reduced cortical cell file number (X.CF) and reduced cortical cell area (CCA), had a reduced total carbon cost (Fig. 10). This reduction in total root carbon cost was associated with increased total root length (Fig. 10), which translated to improved soil exploration and better performance across the eight simulated environments, even though they had contrasting phenotypes for root growth angle and number of nodal roots. Among all phenes of the 96 landraces, reduced cortical cell file number had the greatest contribution to simulated biomass production under isolated stress for drought, nitrogen and phosphorus. The improved performance of clusters 2 and 4, with contrasting root architecture, suggests that the potential disadvantages of the extreme root architecture (e.g. very shallow or very steep) will be compensated for by a reduced cost of soil exploration due to small anatomical phenotypes. Overall, our results suggest that anatomical phenes that reduce the metabolic cost of soil exploration are associated with greater yield stability.

It has been shown that increased lateral branching increases root respiration and root maintenance cost (Zhan *et al*., 2015; Zhan and Lynch, 2015). Our feature selection prediction showed that reduced lateral branching frequency in node 4 would be important for general adaptation across environments (Table 3). However, when we tested the independent effects of each phene state, lateral branching frequency did not confer a significant benefit under nitrogen and phosphorus stress. This can be explained by the relative utility of this phene. For instance, reduced branching frequency was also selected by Boruta for specific synthetic phenotypes for environments *PI608619e*, *PI571439e*, *PI571629e* and *PI571994e* (Table 1). It is important to note that we are testing the effect of lateral branching frequency in node 4 only, while previous studies showed a strong effect on lateral branching frequency when they were evaluated across all axial roots (Postma *et al*., 2014; Rangarajan *et al*., 2018). Further analysis will be needed to determine the effect of lateral branching frequency in each of these environments. This is interesting because it might reflect the trade-offs between the ability of a phenotype to produce greater biomass in a wide variety of environments compared with the ability to produce biomass in its optimal environment (Weiner *et al*., 2021).

**Table 3.** Synthetic integrated phenotypes built based on Boruta and multiple linear regression.

Synthetic general				
BF_N4	CCS	Dia_N5	Dia_N6	
1.27	1434.5	0.33	0.46	
***Synthetic PI571439***				
NRN_N8	NRN_N6	NRN_N7	CCS	BF_N4
11	5	18	1434.5	1.27
***Synthetic PI571523***				
Dia_N5	CCS	Dia_N4		
0.333	1434.5	0.398		
***Synthetic PI571629***				
Dia_N5	BF_N4	Dia_N4	NRN_N5	Dia_N3
0.333	1.27	0.24	5	0.31
***Synthetic PI571913***				
Dia_N5	Dia_N6	Dia_N4	Dia_N7	Dia_N3
0.333	0.76	0.39	0.411	0.313
***Synthetic PI571994***				
Dia_N5	Dia_N4	BF_N4	CCS	
0.333	0.39	1.27	1434.5	
***Synthetic PI608619***				
BF_N4	NRN_N5	CCS	Dia_N5	
1.27	5	1434.5	0.333	
***Synthetic PI516002***				
NRN_N8	NRN_N6	CCS	NRN_N7	AN_N7
1.27	5	1434.5	18	61.9
***Synthetic PI471823e***				
Dia_N5	Dia_N4	Dia_N6	Dia_N3	Dia_N5
0.52	0.24	0.45	0.31	0.52

Dia, root diameter (cm); NRN, nodal root number; AN, root angle (degrees); BF, lateral branching frequency (root/cm); N#, node number; CCS, cortical cell size (µm^2^).

Our results support the hypotheses that root anatomical phenotypes that reduce the metabolic cost of soil exploration and intermediate root architectures will be useful targets of selection for crops with greater yield stability. Previous studies explored the fitness landscape utilizing massive arrays of possible root phenotypes (Rangarajan *et al*., 2018; Ajmera *et al*., 2022), or more refined approaches such as multi-objective evolutionary algorithms (Rangarajan *et al*., 2022) to identify integrated phenotypes adapted to diverse environments. Our work is the first study to explore the utility of different integrated root systems of maize landraces from the Americas across multiple environments *in silico*. This is an example of how functional–structural plant/soil models can be used to identify optimal and stable integrated phenotypes starting from a small population of plants.

## Supplementary Material

mcaf179_Supplementary_Data
