# Supplementary material for: Broad environmental adaptation is associated with root anatomical phenotypes in maize landraces: an in silico study
Source: Ann Bot. 2025 Aug 13;138(2):328–44. doi: 10.1093/aob/mcaf179 (PMC13454703; doi:10.1093/aob/mcaf179)
Supplement: Supplementary file 1 [file mcaf179_supplementary_data.zip › Supplementary.docx]

Supplementary materials for

**Broad environmental adaptation is associated with root anatomical phenotypes in maize landraces: An *in silico* study**

Ivan Lopez-Valdivia^1^, Harini Rangarajan^2^, Miguel Vallebueno-Estrada^3^, Jonathan P. Lynch^1^*

^1^Department of Plant Science, The Pennsylvania State University, University Park, PA, U.S.A., 16802

^2^Department of Plant Biology, University of Illinois Urbana-Champaign, 265 Morrill Hall, MC-116, 505 South Goodwin Ave., Urbana, IL 61801, USA

^3^The LatAmBio Initiative, Irapuato, Guanajuato, Mexico.

**This pdf includes:**

Table S1

Table S1: Origin information of the eight accessions used in this study.

| Accession | Name | Origin | Coordinates | Climatic Zone |
| --- | --- | --- | --- | --- |
| PI 471823 | Costa Rica 139 | Costa Rica | 10.88518100, -85.00984500 | Humid |
| PI 516002 | ARZM 16 001 | Mendoza, Argentina | -33.26666667, -68.15000000 | Arid |
| PI 571439 | Lambayeque 151 | Lambayeque, Peru | -6.70000000, -79.91666667 | Arid |
| PI 571523 | Ica 8 | Ica, Peru | -14.18333333, -75.73333333 | Arid |
| PI 571629 | Cuzco 22 | Cusco, Peru | -12.81666667, -72.71666667 | Sub-humid |
| PI 571913 | Huanuco 191 | Huánuco, Peru | -10.06666667, -75.53333333 | Humid |
| PI 571994 | Madre de Dios 22 | Madre de Dios, Peru | -13.68333333, -69.53333333 | Humid |
| PI 608619 | Minnesota 13 | Colorado, United States | 39.16666667, -108.80000000 | Arid |
